# Online learning resources and social media platforms used by medical students during the COVID-19 pandemic

**DOI:** 10.1186/s12909-023-04906-w

**Published:** 2023-12-19

**Authors:** Samy A. Azer, Deema Alhudaithi, Fay AlBuqami, Haifa AlWaily, Razan AlRabah, Raghad AlKhashan

**Affiliations:** Department of Medical Education, Curriculum Development and Research Unit, College of Medicine, King Saud University, PO Box 2925, Riyadh, 11461 Saudi Arabia

**Keywords:** Online learning, Internet resources, Social media, Technology, Students’ use

## Abstract

**Background:**

The unprecedented COVID-19 pandemic has caused significant disruption to medical students’ education. It imposed challenges that required rapid adaptation to enforced lockdowns and remote learning and changed curriculum delivery from in-person to online learning and virtual technology.

**Objective:**

This study aimed to determine the trends and ratings of using Internet resources and social media platforms by medical students during the COVID-19 pandemic.

**Methods:**

A validated questionnaire was used to explore preferences for Internet resources and social media platforms among undergraduate medical students (years 1, 3, and 5) at King Saud University. The questionnaire comprised three sections- (i) demographic information, (ii) access and use of Internet resources/social media platforms, and (iii) students’ ratings and reasons for using technology-enabled learning during the COVID-19 pandemic.

**Results:**

A total of 320 undergraduate medical students responded to the online questionnaire. The difference in the number of students using the Internet daily across academic years increased significantly as they progressed in the medical course (*p* = 0.025). For learning, YouTube and Videoconferencing (e.g. Zoom) were used by 83.1% and 73.4% of students, respectively, followed by WhatsApp 198 (61.9%). For social interaction, WhatsApp, 310 (96.6%); YouTube, 296 (92.8%); Twitter, 288 (90%); and Zoom, 269 (84.1%) were the platforms used by most students. Regarding concerns about the impact of COVID-19 and social isolation, 250 (78.1%) agreed that technology helped them gain a sense of connectedness to their peers. Over half of students, 187 (58.4%) wished that technologies be integrated more often in their courses, as 245 (76.7%) agreed that it helped engage them with classes.

**Conclusion:**

The study shows that the use of the Internet and social media resources is increasing at all levels to fill the gap in learning and social interaction because of the COVID-19 pandemic. Medical institutions should embrace the effective use of Internet resources and use the experience gained and lessons learned in guiding educators on what type of online resources should be created to add value to students learning even post-pandemic.

## Practical points

- The use of Internet resources and social media platforms has increased for learning and social interaction regardless of the student’s gender or academic year.
- The rate of using some social media platforms by students, such as LinkedIn, was determined by students’ academic year.
- The experience gained and lessons learned should guide educators on what type of online resources should be created even post-pandemic.

## Introduction

Medical schools faced several changes and challenges during the COVID-19 pandemic because of the restrictive laws imposed by public health authorities during 2020/2021 [1]. The facets of medical education have been disturbed, and teaching sessions, including clinical rotations, outpatient clinics, practical classes, and electives, were eliminated and replaced by online and virtual learning. This means total dependence on online resources, virtual education, and video conferencing in curriculum delivery. Therefore, it is time to analyze the changes introduced and the reliance on virtual learning features in the curriculum [2].

The virtual learning environment and online resources enable students to interact with their teachers and their peers anytime and use cutting-edge communication technology to explore their learning needs [3]. Stojan et al. 2022 indicated that online content, ease of access, navigation, and greater interactivity in the learner interface are crucial features of virtual learning [4].

Earlier studies raised concerns about the shift to the online and virtual learning environment, particularly regarding the distraction of students and adverse effects because of learners’ limited visual and auditory channels as outlined in the cognitive load theory of multimedia learning [5]. However, students in the context may have the opportunity to select and use the online learning resources that are most beneficial to their learning and avoid cognitive overload [6–8]. For example, Bostman and Zagenczyk [9] observed that “social media capacity to enable users connect, share and collaborate has made its increasingly common in personal, social, and educational domains. Also students demonstrated interest in using the technologies and social platforms in learning and were able to adopt technologies to replace traditional face-to-face teaching. It appears that learning takes place in their own habitats as opposed to formal classrooms learning. Students also demonstrated that social media learners were able to find answers for their questions through interactions among themselves and the use of social platforms [10].

This approach may necessitate the medical education department and the teachers to introduce means of constructive alignment to foster deep learning using online resources and tailor the mix of these resources appropriately and encourage students to select the educational resources and technology features that they prefer [11, 12].

Therefore, our study aimed to determine the trends and ratings of medical students in years 1, 3 and 5 on using the Internet, social media, and technology resources during the COVID-19 pandemic (November and December 2020).

The knowledge obtained from this research may help medical students expand their learning options and highlight the value of online resources and virtual learning even post-pandemic. The study may also help medical educators tailor their courses according to the learning needs of medical students by incorporating the students’ preferred Internet platforms into the curriculum. We hypothesize that students use the Internet, social media and technology resources equally for entertainment and learning. We also hypothesize that the utilization of technology varies in the number of hours and the preferred platforms across different academic years.

## Methods

### Study design

This cross-sectional questionnaire-based study was conducted among medical students.

### Study setting

King Saud University College of Medicine, Riyadh, Saudi Arabia.

### Participants

Medical students in years 1, 3 and 5 enrolled during the academic year 2020–2021. We have decided to include students from years 1, 3 and 5 because they represent students at the beginning of studying medicine (year 1), middle (year 3 in the program) is the transition year from the preclinical phase to the clinical phase, and the end of their medical education program (year 5), where students are prepared to complete their internship and join the medical workforce. Thus, our data reflect medical students’ overall trends and ratings across the medical program.

### Variables

The variables in this study were the students’ gender, age, and academic year. We aimed to compare these variables against several items in the questionnaire including ownership of a laptop, smartphone, use of the internet, time spent on the internet, and social media platform used.

### Data source measurements

The questionnaire used in this study was adapted from the “Questionnaire on Learner Use of Technology” published in the Technology-Enabled Learning Implementation Handbook, pages 59 to 68 [13]. The principal investigator received approval from the corresponding author to use the questionnaire in this study. Because four questions were found to deviate from the aim of our research, these questions were amended accordingly. The questionnaire comprises three sections; Section A covers the participant’s gender and demographic information; Section B covers the access and use of Internet resources technologies; and Section C covers the rating and reasons for using technology-enabled learning. The questionnaire was converted into an online version using Google Forms. Also, a cover letter explaining the purpose of the study and its aim was included with the questionnaire. The cover letter stated that choosing to be part of this study was voluntary, and the questionnaire was anonymous. Details of the principal researcher were included in the cover letter for any queries about the questionnaire or the study. All participants gave consent before starting to answer the questionnaire.

### Piloting the questionnaire

A pilot study was conducted before the survey was used in the study. We invited ten male and an equal number of female year two students to complete the questionnaire. The responses collected from the participants were analyzed, studied, and comments from these participants were used to amend any unclear wording in the questionnaire [14].

### Data collection and statistical analysis

The data were collected between November and December 2020 in collaboration with the Medical Student College Council. The council facilitated emailing the questionnaire and inviting students in years 1, 3 and 5 to be part of the study. The collected data were converted via Google Forms to an Excel Sheet (Mac version 2016). Two researchers checked that the converted data were accurate and the conversion process did not affect data accuracy [14]. The Statistical Package for the Social Sciences (SPSS version 20.0 statistical software) was used for analyzing the collected data. Numbers and percentages were used for the description of the profiles of the participants. Bivariate statistics were carried out by using a chi-square statistical test, which was employed to look for a link between variables. A *p*-value < 0.05 was considered significant [15].

### Biases

In order to minimize the recall bias in the study, we intended to run the questionnaire during the COVID-19 pandemic (from November to December of 2020).

### Sample size

According to the study “Measuring the extent and nature of use of Social Networking Sites in Medical Education by university students” *P* = 77.6% [16].

We calculated the sample size using this formula [17]:

$$\mathcal{n}= \frac{\mathrm{\rm Z}{\alpha }^{2}\mathrm{\rm P}\left(1-\mathrm{\rm P}\right)}{{\mathcal{d}}^{2}}\mathrm{\,when\, {\rm Z}}\alpha \,=\,1.96\, at\, 95\%\, confidence\, level\, and\, precision\, \left(d\right)\,=\,5\%$$

Our sample size would be 267. In regard to the non-response 20% has been added to the original sample size and the total is 320 students.

### Ethical approval

The study was approved by the Ethical Institutional Review Board (IRB), King Saud University, Riyadh, Saudi Arabia. Approval letter No. E-20-5317, November 2020.

## Results

### Participants’ demographics

In total, we received responses from 320 out of 888 students contacted, a response rate of 36%. Of these, females were 197 (61.6%), and males represented the remaining 38.4%. According to the academic year, participants were 125 (39.1%) from the first year, 123 (38.4%) from the third year, and 72 (22.5%) from the fifth year (Table 1).

**Table 1:** Demographics, device ownership and Internet access during the COVID-19 pandemic

**Gender N (%)**			**Age group N (%)**		**Academic year N (%)**		**Time spent on Internet-related activities N (%)**	
Woman	197 (61.6)		18–19	100 (31.2)	Year 1	125 (39.1)	< 1 h	1 (0.3)
Male	123 (38.4)		20–21	121 (37.8)	Year 2	123 (38.4)	1–2 h	17 (5.3)
			22–23	88 (27.5)	Year 3	72 (22.5)	3–5 h	100 (31.3)
			24–25	10 (3.1)			> 5 h	202 (63.1)
			26–27	1 (0.3)			Do not use daily	-
			> 28	-				
**Devices Ownership N (%)**			**Frequently used devices to access the Internet**^**d**^** N (%)**		**Use of the Internet N (%)**		**Preferred teaching format during the COVID-19 pandemic N (%)**	
Desktop computer	**1**^**a**^	74 (23.1)	Smartphone	287 (89.7)	Daily	311 (97.2)	Traditional face-to-face	156 (48.7)
	**2**^**b**^	231 (72.2)	Tablet	230 (71.9)	Alternate days	1 (0.3)	Completely online	19 (5.9)
	**3**^**c**^	15 (4.7)	Laptop	131 (40.9)	Once a week	5 (1.6)	Blended	145 (45.3)
Laptop	**1**	274 (85.6)	Desktop computer	18 (5.6)	Irregularly	3 (0.9)		
	**2**	26 (8.1)			Rarely	-		
	**3**	20 (6.3)			Never	-		
Smartphone	**1**	316 (98.8)						
	**2**	1 (0.3)						
	**3**	3 (0.9)						
Tablet (e.g., iPad)	**1**	284 (88.7)						
	**2**	21 (6.6)						
	**3**	15 (4.7)						

Keyword: ^a^Yes, ^b^No, but I plan to buy one in the next 12 months, ^c^No, and I do not plan to buy one in the next 12 months, ^d^the question required more than one response

### Device ownership and Internet access

Table 1 shows device ownership. Of nearly all students, 316 (98.8%) owned Smartphones, 284 (88.7%) had Tablet devices, whilst 274 (85.6%) owned Laptops. The least owned device was a Desktop computer, which only 74 (23.1%) of students held. However, 231 (72.2%) of students reported planning to buy a Desktop computer in the next 12 months. Regarding frequency to access the Internet, 287 (89.7%) used Smartphones, 230 (71.9%) used Tablets, and 131 (40.9%) preferred Laptops. Only 18 (5.6%) chose Desktop computers. More than half of the students, 202 (63.1%), spent over 5 h on Internet-related activities, and 100 (31.3%) reported 3–5 h. Only one student said less than 1 h. More than half of the students 187 (58.4%) wished that technologies be integrated more often in their courses, as 245 (76.7%) agreed that it helped in engaging them with classes.

### Social media use in socializing and learning during the COVID-19 pandemic

Table 2 shows that most students, 316 (98.7%), had social media accounts during the pandemic. WhatsApp 310 (96.6%) was the most popular social media platform, followed by YouTube 296 (92.8%) and Snapchat 272 (85%). The least popular platforms were Facebook 53 (16.6%), Goodreads 53 (16.6%), and blogs 19 (5.9%). Regarding social media platforms used for learning purposes, YouTube 266 (83.1%) ranked first, followed by Zoom 235 (73.4%), then WhatsApp 198 (61.9%). Time spent by students on social media showed that nearly half of the participating students, 148 (46.3%), spent 3 to 5 h online. Almost one-third of the students, 124 (38.7%), reported needing to update their social media accounts frequently.

**Table 2:** Social media use in socializing and learning during the COVID-19 pandemic

**Social media account N (%)**				**Time on social media N (%)**			
Yes	316 (98.7)			< 1 h		9 (2.8)	
No	4 (1.2)			1–2 h		66 (20.6)	
				3–5 h		148 (46.3)	
				> 5 h		97 (30.3)	
				Do not use daily		0 (0)	
**Internet resources used in socializing**^**a**^** N (%)**				**Internet resources used in learning**^**a**^** N (%)**			
Blog	19 (5.9)	Telegram	228 (71.5)	Blog	10 (3.1)	Video-conferencing (Zoom)	235 (73.4)
Facebook	53 (16.6)	Twitter	288 (90)	Facebook	12 (3.8)	Online Q-Banks	106 (33.1)
23 (7.2)	53 (16.6)	WhatsApp	310 (96.6)	Goodreads.com	12 (3.8)	Google Charts	39 (12.2)
LinkedIn	55 (17.2)	YouTube	296 (92.8)	LinkedIn	23 (7.2)	Google Calendar	51 (15.9)
Photo sharing (Instagram)	194 (60.6)	Video-conferencing (Zoom)	269 (84.1)	Research Sharing Sites	79 (24.7)	Google Classroom	27 (8.4)
Snapchat	272 (85)			Snapchat	20 (6.2)	Google Docs	189 (59.1)
				Telegram	150 (46.9)	Google Books	190 (59.4)
				Twitter	68 (21.3)	Google translate	172 (53.8)
				WhatsApp	198 (61.9)	Social bookmarking sites (Pinterest)	120 (37.5)
				YouTube	266 (83.1)		

Keyword: ^a^the question required more than one response

### Analyzing data based on gender, age and academic year

Table 3 compares variables concerning gender, age, and academic year. The time spent on the Internet was significantly different (*p* = < 0.001) based on gender but not different based on students’ age (*p* = 0.945) or academic year (*p* = 0.349). Owning a tablet device was significantly different based on age (*p* < 0.001) and academic year (*p* = 0.006), respectively. The relationship was not significantly different based on gender (*p* = 0.071). The difference in the number of students using the Internet daily across academic years increased significantly as they progressed in the medical course (*p* = 0.025). Analysis of other correlations and *p*-values are summarized in Table 3.

**Table 3:** Chi-square *p*-values: comparing variables to gender, age, and academic year

Item	Chi-square *p*-value		
	Gender	Age	Academic year
Owning a desktop	0.002	0.631	0.136
Owning a laptop	0.441	0.835	0.279
Owning a smartphone	0.282	0.835	0.575
Owning a tablet device	0.071	< 0.001	0.006
Use of the Internet	0.323	0.282	0.025
Time spent on the Internet	< 0.001	0.945	0.349
Updating social media status	< 0.001	0.791	0.080
Time spent on social media	0.274	0.606	0.634

### Students rating their computer skills

**Fig. 1:**
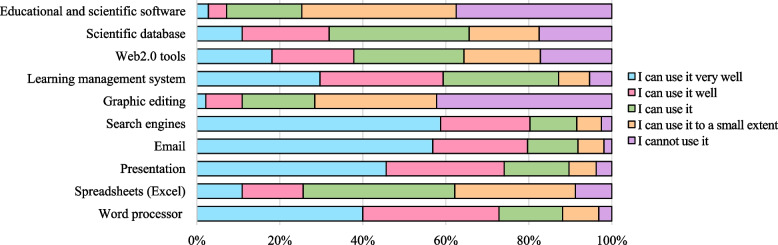
Students rating their skills in computer-related activities

Figure 1 shows more than half of students, 188 (58.8%), reported that they could use search engines very well, and 69 (21.6%) could use them well. Only eight students (2.5%) said they could not use it. Over half of the students, 182 (56.9%) reported they could use email very well, 73 (22.8%) reported they could use it well, and only 6 (1.9%) said they could not use it. Regarding presentation skills, 146 (45.6%) reported they could present their work using technology very well, 91 students (28.4%) could present it well, and only 12 (3.8%) said they could not. The distribution of other skills is summarized in Fig. 1.

### Students rating their reasons for using technology-enabled learning during the COVID-19 pandemic

**Fig. 2:**
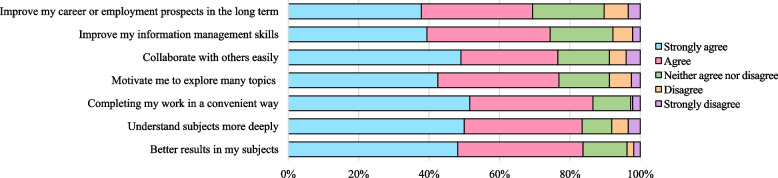
Students rating their reasons for using of technology-enabled learning

Figure 2 shows 165 (51.6%) strongly agreed that the reason for using technology-enabled learning was to help them complete their work in a convenient way, 112 (35%) agreed to the statement, and only 7 (2.2%) strongly disagreed. Half of the students, 160 (50%), strongly agreed that using technology was to understand subjects more deeply, and 107 (33.4%) agreed. Only 11 (3.4%) strongly disagreed. Regarding concerns about the impact of COVID-19 and social isolation, 250 (78.1%) agreed that technology helped them gain a sense of connectedness to their peers. Other reasons for using technology-enabled learning are shown in Fig. 2.

### Students rating the usefulness of computer-related activities during the COVID-19 pandemic

**Fig. 3:**
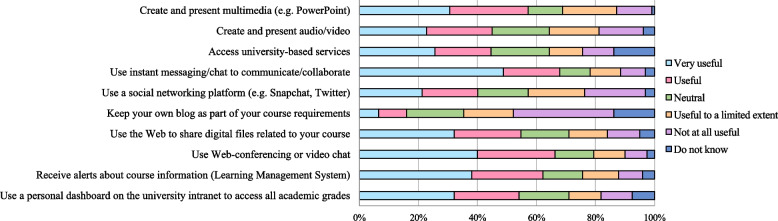
Students rating the usefulness of computer-related activities

Figure 3 shows students’ ratings of the usefulness of each technology. Less than half of the students, 156 (48.8%), found the use of instant messaging/chat to communicate very useful, and 61 (19.1%) reported that it was helpful, while only 27 (8.4%) rated it as not useful. 128 (40%) of students found chatting, web-conferencing, or video very useful, and 84 (26.3%) found it helpful. Only 24 (7.5%) found it not useful at all. 122 (38.1%) found receiving alerts about the course information via the learning management system (LMS) was beneficial, and 77 (24.1%) found it helpful. While 26 (8.1%) found it not useful at all. Other students’ ratings for the usefulness of technology are shown in Fig. 3.

## Discussion

Our study showed that during the COVID-19 pandemic and with the total shift to online and virtual technology to deliver the curriculum, students particularly valued collaborative tools such as WhatsApp groups, web-conferencing or video chatting and virtual learning resources that provide on-demand videos such as YouTube platforms, receiving alerts about the program information via the learning management system.

Our findings showed that students identified several educational benefits of using computer-related activities and online platforms, and they have the skills acquired to use such technologies. The resources that engage students provide indications of the value they attach to those in learning. However, with this shift to the virtual environment and various online learning resources, students may need help integrating the information channeled through integrating motivation to cognitive load [18]. While we have not measured in this study how students manage their cognitive load by being selective in using their online resources in the virtual learning environment, it appears that students were effective in using a broader mix of online learning resources and were able to identify the use of each tool for social interaction or learning most likely was determined by the requirements of the assessment [9]. The assessment method can be one of the deciding factors whether cognitive load becomes intrinsic or extraneous for a task [19].

As seen from the literature, a few years were able to drastically change the way medical students choose to interact with technology. For instance, a 2014 study conducted at the University of Southern Illinois showed that a laptop computer was medical students’ most used technological gadget for personal and learning purposes, followed by a cell phone and smartphone [20]. In contrast to this finding, our study demonstrated that smartphones were the most used, followed by tablets, then laptops devices. Robert Trelease, a medical researcher, described smartphones as a ‘learn anywhere resource’ tool, explaining that students can utilize their smartphones for potential learning anywhere and everywhere [21]. This may be due to the students’ continuous and fast-growing need to access the Internet anywhere and anytime. Another finding of our study was 98.8% of students owned a smartphone device, which could have contributed to the shift in hardware usage as they present an excellent opportunity to always learn due to their wide availability, thus transforming the way medicine is taught and practised [22].

Our study also illustrated that the noticeable shift wasn’t limited to which gadgets students preferred to use but also what social media platforms students used most often. In 2011, Giordano & Giordano using an online survey administered to 644 first-year students and 413 graduate students to investigate their media preferences, found that most students were using Facebook and very few were using Twitter or Linkedin or other social networking sites. Nine years later, our study found that WhatsApp, Twitter, and Snapchat were the most frequently used social media platforms among medical students. Although the study by Giordano & Giordano looked at the social media preferences of first year and graduate students [23], our study focused on medical students throughout the medical program. The data trends indicated that the use of social media platforms is a dynamic change. Students migrate from one Internet technology to another depending on their needs, functionality, and preferences as they progress in the medical program. Our findings agree with Katz and Nandi (2021) study [24]. They found social media platforms such as Facebook, Twitter, Instagram, YouTube, WhatsApp, and Podcasts endow them and help them with different educational purposes in both formal and informal educational settings. Recently, Chambers et al. [25], in a qualitative study, found that social media, particularly Facebook may be a credible platform for delivering online peer-to-peer teaching during the COVID-19 pandemic. The mix of social and professional discussion on the platform was met with caution by the tutors. However, both learners and tutors enjoyed the familiarity of the platform.

As students dive deeper into their medical education journey, they need more flexibility offered by mobile technologies. Thus, these devices become integral to their academic success. Furthermore, our study showed a shift in the daily use of the Internet as students progressed in their academic courses. The scheme and schedules of pre-clerkship years are relatively predictable, and students’ use of online tools reflected this stability. Our study found significant results that prove students from different academic years use social media applications in a way that reflects their educational stage. Our study showed that the most popular social media platform used by year five students (95.8%) was Snapchat. The percentage of students using snapchat in years 3 and 1 were lower, (87.8%) and (76%), respectively. These findings could be related to the experience of students in the clinical years in finding a better social media platform for communication and socializing. The employment-oriented online service LinkedIn was primarily used by fifth-year students (37.5%) in comparison to their younger peers from year 3 (13.8%) and year 1 (8.8%), which demonstrated that senior students were preparing themselves to enter the workforce.

Regarding YouTube, the trend fluctuated as most users were comprised of students from year 3 (97.5%), followed by year 5 (95.8%), and then year 1 (86.4%). An explanation for this pattern of preferences could be that students in their pre-clerkship years rely heavily on textbooks for information. In contrast, students in their clinical years might have already established other sources that better satisfy their clinical education. In contrast, students in their clinical years have more unstructured schedules, and their learning is more spontaneous and opportunistic; as a result, their access to learning resources is integral during clinical rotations. From our results, there is a positive association between the use of the Internet and social media and the student’s progression through the academic years and the learning needs of the students at different stages of the course. Recently, mobile learning (M-learning) devices such as iPad mini, and mobile phone WhatsApp messages were found to have a positive impact on the learning experiences of medical students during their clinical attachments [26–28]. Taken together, our work and current literature support the feasibility of M-learning devices in supporting learning in the clinical environment.

Applying Piaget’s theory and perspective on Socio-Constructivism on cognitive learning in this context when using social media for learning, we may highlight four aspects of Jean Piaget’s model of cognitive development - Maturity, Physical influence, Social environment, and Assimilation/accommodation. This means that knowledge is “socially constructed” and “produced” through the students’ interaction in their environment and the web portals and search engines and through “assimilation” and “accommodation” [29]. In traditional media such as television, radio, movies, and newspapers, the material is prepared by “authorities” or “experts” who decide on the content and distribution. In this way, the majority of users are “consumers”. who cannot contribute to content because it is “one-way traffic” or “centralised”, and there are no mechanisms for interaction, contribution, or modification of content.

Therefore, Jean Piaget’s theory can explain the phenomenon of learning and how cognitive development takes place through the use of social media platforms such as Facebook, Twitter, LinkedIn, and Zoom, commonly used by students during the pandemic. The motivation element for learning is triggered through the learner’s interaction with the environment and the internet [30]. This interaction of the students with the environment for cognitive development has also been supported by Vygotsky [31]. His activity theory assumes that each self-development has a particular intentional activity as a basis, and a learning activity builds on existing learning prerequisites. He also thinks interaction with a more “advanced individual” is more effective than interaction with “a peer”. Alan Bandura’s [32] idea, based on observations, that individual development would be the outcome of a more elaborated behaviour is worth considering. Students’ interaction on social media and debating their views and strength of evidence, outlined in this study, is consistent with these theoretical frameworks outlined here.

The results of this study may encourage program directors and developers of medical curricula to utilize different Internet-based platforms and social media tools to guide students towards better and more efficient learning outcomes [33]. The study is not free from limitations. First, the results represent students’ views from one institute in the country. A more considerable study covering major universities may present an ideal picture. However, King Saud University is the top university in the kingdom, and the data collected from this study represent students’ views throughout the medical program. Second, as with all questionnaires, the information collected about ratings and trends does not represent objective measures. Yet, the questionnaire is an ideal tool for collecting and managing data about students’ trends and uses of online resources from this number of participants. Third, although the study covers the students’ responses from years 1, 2, and 3, the number of responses was relatively low.

## Conclusion

The study depicts the trends and ratings of medical students during the COVID-19 pandemic on using the Internet and, social media platforms across the medical program. A uniform pattern of using technology devices was seen among students for learning and social interaction regardless of gender or academic level. Moreover, the changes in the trends of using the Internet and social media platforms by students as they progress in the academic program may reflect a progressive shift in the type of technology platforms that suit their learning needs and leisure time. The pandemic should be taken as a stimulus and an opportunity for medical and health colleges to create more online resources even post-pandemic. The experience gained and the lessons learned should be used in guiding educators on what type of online resources should be created to add value to the student’s learning.

## Data Availability

The datasets used and analysed during this study are available from the corresponding author on reasonable request.
